# Microglial CR3-mediated synaptic pruning in the dmPFC promotes the generation and maintenance of chronic muscle pain via glutamatergic dysfunction

**DOI:** 10.1038/s12276-026-01666-7

**Published:** 2026-03-02

**Authors:** Meiling Luo, Likai Wang, Yanan Liang, Qianxi Xu, Siqi Zhang, Xiangxin Xing, Shuangyang Niu, Yonghui Wang

**Affiliations:** Rehabilitation Center, Qilu Hospital of Shandong University, Jinan, China

**Keywords:** Synaptic plasticity, Inflammation

## Abstract

Chronic muscle pain (CMP) is highly prevalent, frequently comorbid with emotional disorders and characterized by a high risk of recurrence. Yet, the complex mechanisms underlying the generation and maintenance of CMP remain unclear, limiting the development of therapy. Here we identified suppressed glutamatergic neuronal excitability and reduced synaptic plasticity in the dorsomedial prefrontal cortex (dmPFC) of CMP rats using fiber photometry, patch-clamp, in vivo recording of field potentials and other techniques. The optochemical genetical activation of dmPFC glutamatergic neurons alleviated pain and anxiety-like behaviors. Single-cell RNA sequencing revealed a marked upregulation of proinflammatory microglia and complement receptor 3 (CR3) in the dmPFC, which correlated with reduced neuronal excitability and synaptic function. Flow cytometry and immunofluorescence further showed that hyperactive glutamatergic neurons induced microglial activation, proliferation, polarization and chemotaxis. Notably, the inhibition of microglia or knockdown of microglial CR3 restored dmPFC glutamatergic neuronal excitability and synaptic plasticity, thereby alleviating hyperalgesia and anxiety-like behaviors. This study demonstrates that microglial CR3-dependent synaptic pruning underlies suppressed glutamatergic neuronal excitability and reduced synaptic plasticity, playing a pivotal role in CMP generation and maintenance. These findings uncover novel microglia–neuron interactions and offer promising therapeutic targets for CMP and its emotional comorbid disorders.

**Figure:**
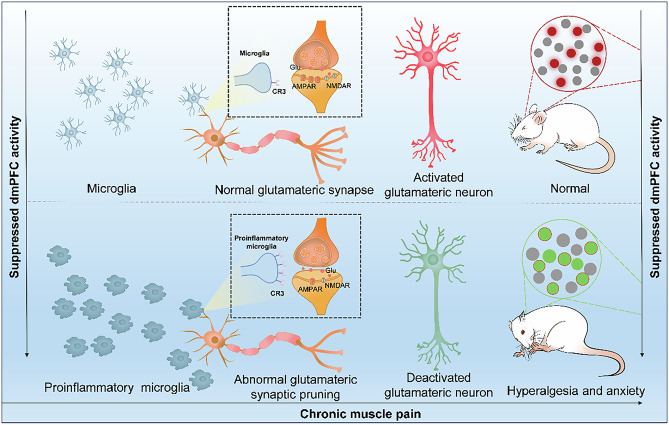
Microglial CR3-mediated synaptic pruning in the dmPFC suppresses glutamatergic neuronal excitability, contributing to chronic muscle pain generation and maintenance and represents a promising therapeutic target.

Microglial CR3-mediated synaptic pruning in the dmPFC suppresses glutamatergic neuronal excitability, contributing to chronic muscle pain generation and maintenance and represents a promising therapeutic target.

## Microglial CR3 pruning links pain and neuronal excitability

Chronic muscle pain (CMP) affects many people worldwide, often leading to emotional issues such as anxiety. Current treatments offer only temporary relief, suggesting that deeper brain mechanisms are involved. Researchers explored how the brain’s dorsomedial prefrontal cortex (dmPFC) is involved in CMP. They used a rat model to study this, using techniques such as optogenetics and chemogenetics. They found that reduced activity in the dmPFC is linked to CMP. This reduction is due to microglial cells pruning synapses, which decreases neuron activity. By restoring neuron activity in the dmPFC, they alleviated pain and anxiety in rats. The study suggests that targeting microglial activity could be a new way to treat CMP. Future research could explore how these findings apply to humans and whether similar treatments could be developed for chronic pain relief.

This summary was initially drafted using artificial intelligence, then revised and fact-checked by the author.

## Introduction

Chronic musculoskeletal pain is the most prevalent form of chronic pain, which affects over 40% of the global population and poses health and socioeconomic burden^1^. Among these, chronic muscle pain (CMP) is particularly typical, arising from chronic muscle fatigue, injury and dysregulated neuromodulation. Characterized by prolonged duration and frequent recurrence, CMP severely impairs patients’ quality of life and is frequently associated with emotional disorders such as anxiety^2^. The current clinical management of CMP primarily involves pharmacological interventions and physical therapy. Although effective for transient symptom relief, their limited ability to prevent relapse implies underlying sustained central mechanisms^3–5^. Therefore, elucidating the pathological basis of CMP and identifying intervention targets to prevent its generation and maintenance are critical challenges in chronic pain research.

The dorsomedial prefrontal cortex (dmPFC) has emerged as a central hub for the emotional and affective dimensions of pain^6,7^. In chronic inflammatory pain models, reduced dmPFC neuronal excitability has been linked to pain sensitization and emotional dysregulation^8^. Paradoxically, other chronic pain conditions feature hyperexcitable dmPFC neurons that can induce pain- and anxiety-like behaviors^6^. This apparent discrepancy highlights the complex and dynamic role of excitatory and inhibitory neuronal activity in the dmPFC. Notably, synaptic plasticity forms the foundation of neuronal excitability and network dynamics^9,10^. Researchers have found that synaptic plasticity changes in the brain directly contribute to chronic pain, manifesting as structural and functional synaptic modifications that may serve as neural biomarkers^11^. Structural synaptic plasticity involves alterations in synapse number, morphology, whereas functional plasticity is often reflected in long-term potentiation (LTP) triggered by the activated NMDA receptor and enhanced AMPA receptor^9^. These changes not only modify local excitability but also contribute to the persistence of pain signaling. However, the changes and the cellular and molecular mechanisms underlying synaptic plasticity in CMP remain unexplored.

Glial cells have long been regarded as passive structural and metabolic supporters of neurons in the central nervous system and actively modulate neuronal function and synaptic plasticity. These glia cells exhibit dynamic bidirectional communication with neurons and are actively involved in synaptic pruning, plasticity modulation and neuroinflammation^12,13^. In the context of chronic pain, neuron–glia interactions maintain neuronal homeostasis and regulate synaptic plasticity^14,15^. Aberrant neuronal activity can trigger glial activation, including chemotaxis, proliferation and polarization. In turn, activated glial cells release cytokines and neurotrophic factors that feed back to modulate neuronal excitability and synaptic function^16^. Despite these advances, the neuron–glia interaction underlying excitability changes and synaptic plasticity in the dmPFC of CMP rats remains poorly understood.

In this study, using optogenetics, chemogenetics, electrophysiology and imaging, we first establish that reduced synaptic plasticity in the dmPFC drives CMP, mediated by microglial CR3-dependent synaptic pruning that suppresses glutamatergic neuronal excitability. CMP rats model exhibited markedly reduced dmPFC glutamatergic activity and synaptic plasticity, whereas restoring the excitability of dmPFC neurons notably alleviated mechanical hypersensitivity and anxiety-like behaviors. Single-cell RNA (scRNA) sequencing revealed a pronounced upregulation of proinflammatory microglia and CR3 expression in the dmPFC, closely associated with synaptic dysfunction. The knockdown of microglial CR3 reversed synaptic plasticity, restored excitatory neuronal activity and attenuated both pain and emotional symptoms. Collectively, our findings uncover a previously unrecognized role of microglial CR3-mediated synaptic pruning in driving glutamatergic dysfunction during chronic pain, offering a promising therapeutic target for CMP and its affective comorbidities.

## Materials and methods

### Animals

The experiments were conducted using adult male and female Sprague–Dawley rats (6–8 weeks of age, weighing 160–200 g), housed in clear plastic cages under a 12-h light–12-h dark cycle. Animals had ad libitum access to food and water and were maintained in a controlled environment with a constant temperature of 23 °C and humidity of 50% ± 10%. All rats were procured from Ji’nan Pengyue Laboratory Animal Breeding. All experimental procedures were approved by the Animal Care and Use Committee of Qilu hospital of Shandong University (approval number: (KYLL-2021(KS)-536) and KYLL-2024(ZM)-511), according to the guidelines of the International Association for the Study of Pain.

### CMP model

Following animal anesthesia (intraperitoneal administration of 1% sodium pentobarbitone (40 mg/kg)), as elsewhere^17–20^, a volume of 0.1 ml 5.8% hypertonic saline was intramuscularly injected into the left gastrocnemius muscles (GS) in rats to establish muscle nociception. The injection site was located at the middle part of the ipsilateral GS muscle, and the depth of the needle insert into the GS muscle was about 0.5 cm. The injection procedure was performed manually and lasted more than 30 s. As control, a volume of 0.1 ml 0.9% saline was intramuscularly injected at the designated location. Supported by magnetic resonance imaging (MRI) and ultrasound imaging, the post-modeling time points of 14 days, 28 days and beyond in rat muscle pain (MP) models were defined as CMP phase.

### MRI

Rats were allowed an additional 30 min to establish stable physiological parameters before functional MRI (fMRI) scanning^21^.

### PWT and PWLs

All behavioral assessments were conducted in awake, unrestrained, age-matched rats. Habituated rats were subjected to behavioral testing by an observer blinded to group identity.

Mechanical hypersensitivity was assessed using von Frey filaments (0.008–300 g) applied to the hind paw plantar surface. The 50% paw withdrawal threshold (PWT) was calculated via the up–down method using the formula: 50% PWT = 10^(*Xf* *+* *kδ*)^/10,000, where Xf represents the final filament value, *k* the response–pattern coefficient and *δ* the log-interval between filaments.

Paw withdrawal latencies (PWLs) to thermal stimulus were measured by a focused radiant heat (20 W of power) applied to the injured hind paw of the animals. PWLs were recorded five times and averaged as the thermal pain threshold.

### OFT

Rats were placed in a 100 cm × 100 cm × 40 cm open field for 8 min while locomotor activity (total distance traveled) and anxiety-like behavior (central zone time/distance) were automatically tracked (Smart 3.0 software). All animals were habituated to the testing room for 2–3 h pre-assessment.

### Stereotaxic injection

Rats were anesthetized (1% sodium pentobarbital, 40 mg/kg intraperitoneally) and stereotaxically injected with 0.75 μl viral vectors or tracers (BDA/FG) into dmPFC (anteroposterior (AP) + 3 mm, mediolateral (ML) ± 0.5 mm, dorsoventral (DV) −4.3 mm) and ventrolateral periaqueductal gray (vlPAG) AP −7.8 mm, ML ± 0.8 mm, DV 6 mm at 60 nl/min. Needles remained for 10 min post injection to prevent reflux. Viral-injected animals recovered for 2–3 weeks before experiments. The viruses were all purchased from Shanghai Genechem. BDA was purchased from Thermo Fisher (N7167, 0.1 g/ml), and FG was purchased from Fluorchrome (780000, 0.04 g/ml).

### Fiber optic ferrules or cannula implantation

For in vivo calcium imaging, pAAV-hSyn-GCaMP6s was injected into dmPFC followed by GRIN lens implantation (DV 4.1 mm), secured with dental cement. For pharmacological interventions, guide cannulas were implanted in lateral ventricles (AP −1 mm; ML ±1.5 mm; DV 3.5 mm) for minocycline (MINO) administration. After a 1-week recovery, the neuronal activity was recorded during behavioral tests using fiber photometry (RWD Life Science).

### NMT (Xuyue Company)

For the transmembrane transport rate and direction of Ca^2+^ in the dmPFC, vlPAG or spinal dorsal horn (SDH) slice, 300 µm was detected by noninvasive microtest technology (NMT). The Ca^2+^ flux microsensor was positioned under the microscope just above the locus (brain slice) to be measured, and the detection was started and recorded for 8 min, with six replicates detected in each group. The Fluxes data were directly read by imFluxes V3.0 software (Xuyue 2), and the flux unit was picomole cm^−^^2 ^s^−1^, with a positive value representing efflux and a negative value representing absorption.

### Chemogenetic test

The rats, following virus injection, received an intraperitoneal injection of clozapine-*N*-oxide (CNO; C879476, 1 mg/kg/d, MCE) dissolved in dimethyl sulfoxide (DMSO) and then diluted with 0.9% saline to a final concentration of 1 mg/ml in 5% DMSO solution, followed by the detection of the pain thresholds and the recording of pain-like behaviors.

### Optogenetic test

For optogenetic manipulation, pAAV-CaMKIIa-ChR2-mCherry (activation) or pAAV-CaMKIIa-eNpHR3.0-EYFP (inhibition) viruses were injected into dmPFC with optical fiber implantation (0.2 mm above injection site). After a 3-week expression, MP models were established (28-day duration) with behavioral testing at day 14 post induction. The activation used 473-nm light (20 Hz, 5 ms pulses, 15 mW), whereas inhibition used 589-nm light (same parameters). The CR3 knockdown was combined with inhibition experiments.

### In vivo electrophysiological recording of field potentials

To study the synaptic strength of the medial dorsal thalamus (MDT)–dmPFC pathway, we recorded optical field excitatory postsynaptic potential (fEPSP) in the dmPFC after injecting AAV2/9-CamKIIa-oChIEF-tdTomato into the MDT. At 4 weeks after viral injection, animals were anesthetized again with 1% sodium pentobarbital (100 mg/kg body weight) and fixed in a stereotaxic frame (RWD Instruments).

An eight-channel electrode and an optical fiber in the center (0.4 mm above the tip of the electrodes), which was lowered into the brain targeting the right dmPFC (AP +3 mm from bregma, ML −0.5 mm from the midline, DV −3 mm from the midline). Rats were single-housed for 7 days for recovery after surgery, and optical fEPSPs were then recorded in the homecage. For optical stimulation, the optical fiber was connected to a 473-nm laser. After a stable baseline was established for at least 15 min, optical high-frequency stimulation (eight trains of 150 × 2-ms pulses at 150 Hz, 19-s intertrain interval) was delivered at the recording site, followed by fEPSP recordings for at least 50 mins. Each fEPSP was normalized to field potentials 15 ms before optical stimulation.

### Immunohistochemical and mIHC

Transverse sections (8–10 μm) of the forebrain containing dmPFC, vlPAG or SDH: after warming to room temperature for about 1 h and PBS washes, the sections were air-dried and circled with a histological pen. Sections were blocked with 5% BSA for 30–60 min before overnight incubation with primary antibodies at 4 °C. After PBS washes, immunofluorescent secondary antibodies diluted in 1% BSA were applied for 45 min at 37 °C, followed by DAPI and mounted with antifade solution. Primary antibodies included mCherry (1:1,500, Thermo Fisher), GFP (1:2000, Abcam), NeuN (1:400, Abcam), cFos (1:1000, R&D Systems), Iba1 (1:500, Abcam), SYN (1:500, Abcam) and DAPI (10 μg/ml, Sigma). Secondary antibodies (1:500, Jackson Laboratory) were conjugated to fluorescein, Alexa Fluor-488, Alexa Fluor-594 or Alexa Fluor-647.

Multiplex immunohistochemistry (mIHC) was performed on 4-μm FFPE sections using sequential Opal fluorophore labeling (Akoya Biosciences) with tyramide signal amplification. Three antibody panels targeted: panel 1: (PSD95/CR3/Iba1/NeuN/cFos/CD86); panel 2: (cFos/VgluT2/GAD65&67); and panel 3: (SYN/VgluT2/GAD65&67). The sections were counterstained with DAPI and imaged. For phagocytosis analysis, IMARIS-generated three-dimensional (3D) microglial surfaces were used to quantify internalized PSD95^+^/CR3^+^ puncta via threshold-based reconstruction and ‘Spots’ detection.

### WB

The dmPFC, vlPAG or L4–L5 spinal cord segments were dissected and placed in Eppendorf (EP) tubes. Primary antibodies against pCREB (1:5000, Abcam), PSD95 (1:1000, Abcam), β-actin (1:5000, Proteintech) and CR3 (1:2,000, Abcam) were incubated overnight at 4 °C. After washing, membranes were incubated with anti-rabbit or anti-mouse secondary antibodies (1:5000, Sparkjade) at room temperature for 1 h. The blots were visualized using BeyoECL Plus (Beyotime), and image intensity was analyzed with a Fusion imaging system.

### Quantitative real-time PCR

Cortical tissues from the dmPFC were collected for total mRNA extraction (*n* = 6 rats per group). GAPDH was used as the internal reference, and cycle threshold (CT) values were analyzed using the 2^−ΔΔCT^ method. Gene-specific primers were obtained from Tsingke Biotechnology (Supplementary Table 1).

### Flowcytometry

Bilateral dmPFC were isolated from the brain and homogenized into single-cell suspensions, followed by isolation on a Percoll density gradient as previously described. Flow cytometry analysis revealed that approximately 90% of the cells recovered from this Percoll interphase were CD45^int-^CD11b^+^ microglia. The isolated microglia were then labeled with fluorescein isothiocyanate AF700 anti-rat CD45 (2 μg/ml; BioLegend), Percp-cy5.5 anti-rat CD11b (5 μg/ml; Biosis), FITC anti-rat CD38 (1 μg/ml; BioLegend), PE anti-rat CD86 (BioLegend), BV421 anti-rat CD206 (5 μg/ml; Biosis) and antibodies for 30 min at room temperature. Data were collected using a four-laser Becton Dickinson FACS Calibur (BD Biosciences) and analyzed using FlowJo software.

### TEM

The dmPFC tissues were dissected and rapidly isolated and immersed in 4% glutaraldehyde at 4 °C overnight. Then, tissues were cut into three 1-mm blocks with a sharp blade and sent to Shandong University for subsequent fixation, embedding, slicing and staining. On the basis of three classical criteria for determining excitatory asymmetric synapses: (1) round vesicles, (2) dense postsynaptic density (PSD) and (3) wide synaptic cleft, asymmetric synapses were selected for analysis in this study^22^.

### Electrophysiology

Coronal brain slices containing the dmPFC were prepared as previously described. Transverse slices (300-μm thick) were obtained using a vibrating microtome (Leica VT 1200s) at 4 °C and placed in a submerged chamber with artificial cerebrospinal fluid (124 mM NaCl, 2.5 mM KCl, 2 mM CaCl_2_, 2 mM MgSO_4_, 25 mM NaHCO_3_, 1 mM NaH_2_PO_4_, 10 mM glucose, 1 mM ascorbate and 3 mM sodium pyruvate). Recordings were performed in voltage-clamp or current-clamp mode using the Axon 700B amplifier (Axon Instruments), with data acquisition and analysis facilitated by patch-clamp software (version 10.02, Axon Instruments). Thus, we were able to measure stable extracellular AP firing for hours. The recording pipette was filled with intracellular solution containing in mM: 140 potassium gluconate, 5 KCl, 10 HEPES, 0.2 EGTA, 2 MgCl_2_, 4 MgATP, 0.3 Na_2_GTP and 10 Na_2_-phosphocreatine (pH 7.3 with KOH). In addition, we recorded electrical stimulation-evoked excitatory postsynaptic currents (eEPSCs) or electrical stimulation-evoked inhibitory postsynaptic currents (eIPSCs) in the absence of TTX to determine whether JWH133 alters eEPSCs or eIPSCs in dmPFC neurons. For glutamate receptor-mediated currents, neurons were clamped at 40 mV, and picrotoxin (PTX, 100 μM) was added to block GABA_A_ receptor-mediated inhibition. The NMDA receptor antagonist D-APV (50 μM) was then bath-applied for 10 min to isolate AMPA receptor-mediated excitatory postsynaptic currents (EPSCs). The AMPA-mediated EPSCs were digitally subtracted from total evEPSCs to define NMDA-mediated EPSCs. The AMPA/NMDA ratio was used as a measure of glutamatergic synaptic plasticity^23^. The electrophysiological data recorded in this study were initially processed using Clampfit 10.8 offline.

### Single-cell sequencing and analysis

#### Tissue dissociation and preparation

Fresh dmPFC tissues were stored in sCelLive Tissue Preservation Solution (Singleron) on ice within 30 min post surgery. Then, they were digested and processed into cell suspension. The mixture was centrifuged at 300*g* for 5 min at 4 °C, and the pellet was resuspended in PBS. Samples were then stained with trypan blue for the cell viability assessment.

#### RT, amplification, library construction and primary analysis of raw read data (scRNA-seq)

Single-cell suspensions (2 × 10^5 ^cells/ml) in PBS (HyClone) were loaded onto a microwell chip using the Singleron Matrix Single Cell Processing System. scRNA sequencing (scRNA-seq) libraries were constructed following the GEXSCOPE Single Cell RNA Library Kits protocol (Singleron). Raw reads were processed to create gene expression profiles using CeleScope v1.15.0 (Singleron Biotechnologies) with default settings.

#### A series of bioinformatic analysis

Quality control, dimension reduction and clustering (Scanpy), batch effect removal, differentially expressed genes analysis (Scanpy), pathway enrichment analysis, cell type annotation, pseudotime trajectory analysis: monocle2, functional gene module analysis (Hotspot), UCell gene set scoring and transcription factor regulatory network analysis were performed.

### Statistical analysis

Data were represented as mean (s.e.m.). Single-variable comparisons were made with two-tail paired or unpaired Student’s *t*-tests. Group comparisons were made using either one-way or two-way analysis of variance (ANOVA) followed by post hoc tests in Graphpad Prism 9.0.0. (121). Statistical significance is exhibited as *P < 0.05; **P < 0.01; ***P < 0.001; ****P < 0.0001; ns, not significant.

## Results

### Suppressed dmPFC is found in CMP

**Fig. 1:**
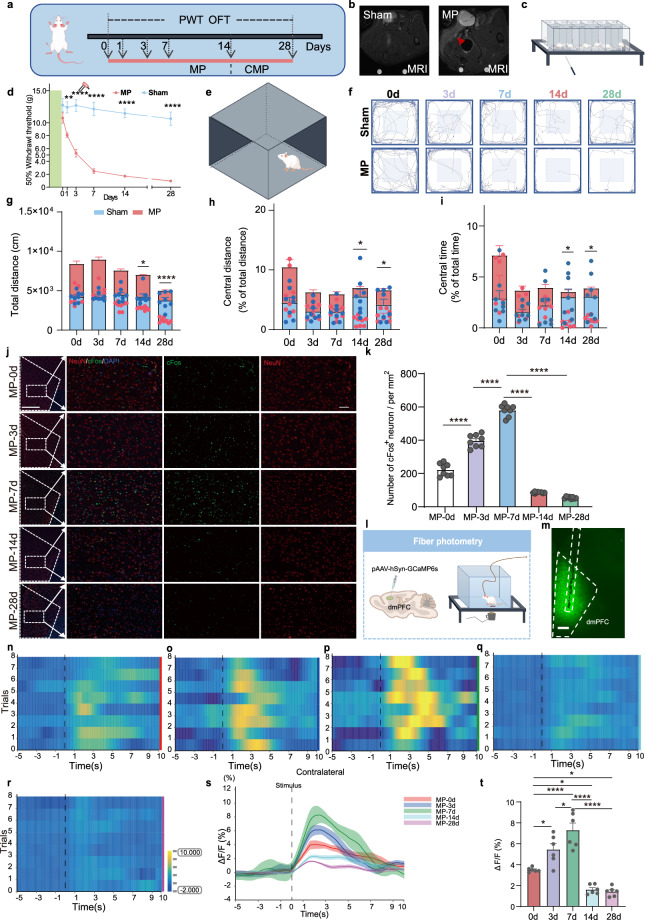
The dmPFC is suppressed in rats with CMP. **a** An experimental schematic for the CMP model and behavioral tests. **b** The MRI images of the rat GS muscle; the arrows point to the tissue inflammation. **c** A schematic of von Frey’s mechanical pain test. **d** The mechanical thresholds of ipsilateral hind paws post MP or sham injury (*n* = 10; two-way ANOVA with Bonferroni’s post hoc test; *MP versus sham). **e** A schematic of exploratory behavior in the OFT. **f** A representative OFT trace. **g**–**i** The total distance traveled (**g**) percent central distance (**h**) and percent time spent in the central area (**i**) quantified over five time points (*n* = 8, two-tailed paired or unpaired Student’s *t*-test). **j** The IF images showing the colabeling of neurons and cFos in dmPFC. Scale bar, 500 mm and 50 mm (enlarged insets). **k** The quantification of cFos^+^ neurons number (*n* = 4 rats, two-tailed unpaired Student’s *t*-test). **l** A schematic of pAAV-hSyn-GCaMP6s injection. **m** The fiber photometry recordings with confocal image showing viral expression (green) and optical fiber tracks. Nuclei are stained blue (DAPI). Scale bar, 500 µm. **n**–**r** A heat map of dmPFC neuronal responses to contralateral hind paw stimuli, and panels **n**–**r** correspond to the MP group at 0, 3, 7, 14, and 28 days, respectively. **s** A representative photometric traces. **t** The quantification of GCaMP6s signals (*n* = 6; 8 trials per animal; one-way ANOVA with Bonferroni’s post hoc test). Statistical significance is exhibited as *P < 0.05; **P < 0.01; ***P < 0.001; ****P < 0.0001; ns, not significant.

To elucidate the alterations in neuronal excitability within the dmPFC of rats with CMP, we conducted a comprehensive set of targeted experiments (Fig. 1a). First, we verified tissue inflammation in gastrocnemius muscle, a hallmark of CMP, confirmed by MRI to validate our model (Fig. 1b). For developmental pain behavior assessment, rats in the MP group exhibited an obvious reduction in mechanical thresholds within the first 7 days, with a gradual decrease from day 14 to 28 in injured hind paws (Fig. 1c, d). We also performed thermal stimulation; however, complex changes were observed for the first 7 days, with persistent pain perception slightly intensifying from day 14 to 28 after MP (Supplementary Fig. 1a, b). Considering the sensory to emotional–cognitive progression of pain, the open field test (OFT) was also evaluated (Fig. 1e). The MP animals showed a gradual decrease in travel distance, notably in total distance on day 14 and 28 and in central distance ratio traveled 14 days post injury (Fig. 1f–h). In addition, the percentage of time spent in the center of the OFT markedly decreased by day 14 post injury and remained low through day 28 (Fig. 1i). These results show that rats exhibited pain sensitization and accompanied by pain-associated anxiety-like behaviors when suffering from CMP.

Functional and molecular analyses revealed the time-dependent modulation of dmPFC activity during CMP progression. The initial fMRI showed reduced dmPFC excitability in CMP rats (Supplementary Fig. 1c). The cFos immunohistochemistry demonstrated biphasic changes: increased neuronal activation at 3–7 days post MP followed by strong suppression at 14–28 days (Fig. 1j, k). The in vivo fiber photometry of GCaMP6s-expressing neurons (Fig. 1l, m) confirmed this temporal pattern, showing enhanced calcium transients in response to noxious thermal stimuli during early phase (3–7 days) but blunted responses in chronic-phase (14–28 days) (Fig. 1n–t). NMT provided complementary electrophysiological evidence: the acute phase (3–7 days) exhibited increased Ca^2+^ influx (hyperexcitability), whereas the chronic-phase showed predominant Ca^2+^ efflux (hypoexcitability) (Supplementary Fig. 1d, e). The above results suggest that the dmPFC is suppressed in the CMP stage.

### Suppressed excitability of glutamatergic neurons in the dmPFC drives to the generation and maintenance of CMP

**Fig. 2:**
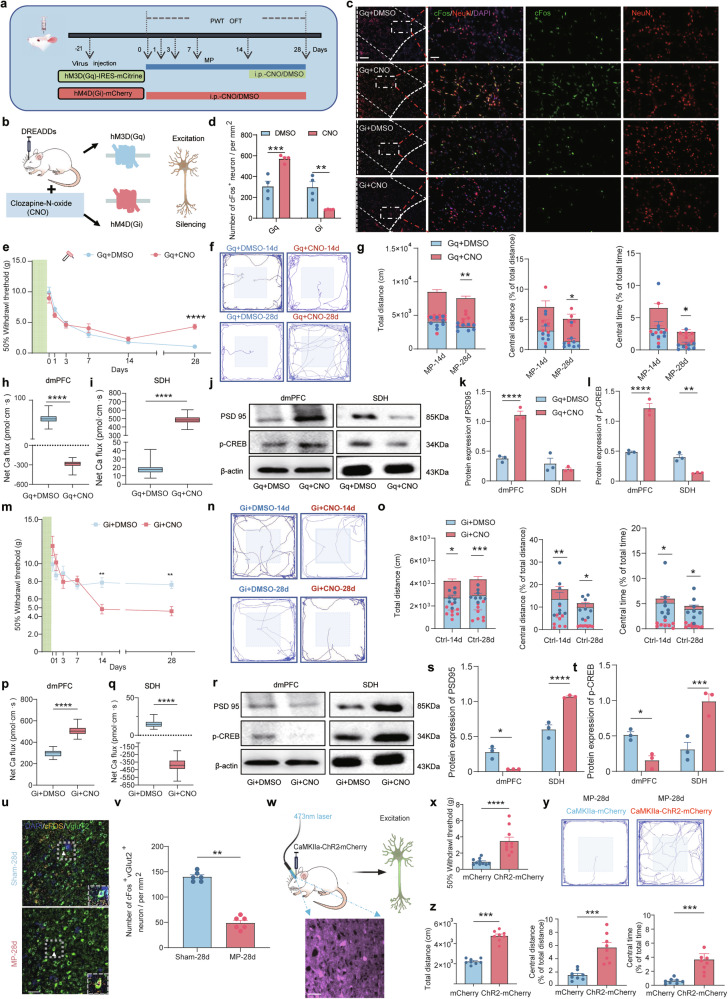
Suppressed glutamatergic excitability in the dmPFC drives CMP. **a** The experimental schematic diagrams of hM3D(Gq) and hM4D(Gi) for CNO administration. **b** A schematic of DREADDs regulation of dmPFC activity. **c** The IF images showing the colabeling of neurons and cFos in dmPFC. Scale bar, 500 mm and 100 mm (enlarged insets). **d** The quantification of cFos^+^ neurons number (*n* = 4 rats, two-tailed unpaired Student’s *t*-test). **e** The mechanical thresholds post hM3D(Gq) activation (*n* = 10; two-way ANOVA with Bonferroni’s post hoc test). **f** A representative OFT trace. **g** The quantification of OFT results after DREADDs administration (*n* = 8, two-tailed unpaired Student’s *t*-test). **h** The statistical analysis of NMT recordings in the dmPFC following hM3D(Gq) regulation, (*n* = 6, two-tailed unpaired Student’s *t*-test). **i** The NMT recordings in the SDH after hM3D(Gq) regulation (*n* = 6, two-tailed unpaired Student’s *t*-test). **j** The representative WB images of dmPFC and SDH. **k**, **l** The quantification of PSD95 (**k**) and pCREB (**l**) protein (*n* = 3 from six rats, one-way ANOVA with Bonferroni’s post hoc test). **m** Mechanical thresholds post hM4D(Gi) activation (*n* = 10; two-way ANOVA with Bonferroni’s post hoc test). **n** A representative OFT trace. **o** The quantification of OFT results after hM4D(Gi) administration (*n* = 8, two-tailed unpaired Student’s *t*-test). **p** NMT recordings in the dmPFC following hM4D(Gi) regulation (*n* = 6, two-tailed unpaired Student’s *t*-test). **q** NMT recordings in the SDH after hM4D(Gi) regulation (*n* = 6, two-tailed unpaired Student’s *t*-test). **r** Representative WB images of dmPFC and SDH. **s**, **t** The quantification of PSD95 (**s**) and pCREB (**t**) protein (*n* = 3 from six rats, one-way ANOVA with Bonferroni’s post hoc test). **u** The IF images showing the colabeling of VgluT2 and cFos in dmPFC. Scale bar, 50 mm. **v** The quantification of VgluT2^+^ and cFos^+^ neuron numbers (*n* = 6 rats, two-tailed unpaired Student’s *t*-test). **w** A schematic of pAAV-CaMKIIa-GCaMP6s injection. **x** The mechanical threshold after activating glutamatergic neurons within dmPFC. **y** A representative OFT trace. **z** Left: the quantification of total distance traveled in the open field. Middle: the percentage of the distance traveled in the central area to total distance in the open field. Right: the percentage of the time spent in central area to total time spent in the open field. *n* = 8, two-tailed unpaired Student’s *t*-test. Statistical significance is exhibited as *P < 0.05; **P < 0.01; ***P < 0.001; ****P < 0.0001; ns, not significant.

To further investigate the role of dmPFC excitability in the development of CMP, we then introduced DREADDs in this part. Rats received infusions of either pAAV-hSyn-hM3DGq-IRES-mCitren or pAAV-hSyn-hM4DGi-mCherry into the dmPFC 21 days before injury (Fig. 2a). The expression of 3D(Gq) and 4D(Gi) receptors in the dmPFC allows the selective activation or inhibition of neuronal activity through intraperitoneal administration of CNO (Fig. 2b–d). Effective viral transfection was confirmed, and the activation/inhibition capabilities were assessed by immunofluorescent staining (Supplementary Fig. 2a). In the Gq+CNO group, 14-day activating dmPFC neurons can result in an elevated noxious mechanical pain threshold in CMP group on day 28 (Fig. 2e). In OFT, animals in the 3D(Gq) group treated with CNO showed increased total distance traveled, central distance and time spent in the central area in CMP group on day 28 (Fig. 2f, g). These results demonstrated that the activation of the dmPFC can decrease allodynia and comorbid anxiety-like behaviors in rats with CMP.

Given the dmPFC’s influence on downstream sensory pathways via descending pain modulation, we investigated its role in SDH neuronal activity during CMP development. Immunofluorescence (IF) revealed substantially elevated cFos expression in SDH under CMP conditions (Supplementary Fig. 2b, c), where similar trends can be seen in the increased expression level of pCREB and PSD95 in dmPFC (Supplementary Fig. 2d–f). The bidirectional modulation of dmPFC activity produced opposing effects on SDH cFos expression (Supplementary Fig. 2g–i), demonstrating the dmPFC-mediated regulation of SDH excitability in CMP progression. The chemogenetic activation of dmPFC (Fig. 2h) suppressed the SDH activity (Fig. 2i), with corresponding decreases in pCREB and PSD95 expression (Fig. 2j–l), indicating that dmPFC activation alleviates CMP by normalizing SDH hyperexcitability. Conversely, 14-day dmPFC inhibition in wild-type animals reduced mechanical pain thresholds (Fig. 2m) and decreased locomotor activity (Fig. 2n, o). This intervention (Fig. 2p) enhanced SDH activity (Fig. 2q) while reciprocally altering pCREB and PSD95 expression in dmPFC versus SDH (Fig. 2r–t). We identified functional bilateral projections between dmPFC and vlPAG (Supplementary Fig. 2j). CMP decreased vlPAG but increased SDH activity (Supplementary Fig. 2k), with NMT confirming their reciprocal activation (Supplementary Fig. 2l). Coordinated changes in PSD95 and pCREB (Supplementary Fig. 2m–p) supported dmPFC’s positive regulation of vlPAG, suggesting a potential dmPFC → vlPAG → SDH pain control circuit (Supplementary Fig. 2q). Collectively, these findings demonstrate that dmPFC hypoactivity drives CMP pathogenesis by promoting SDH neuronal hyperexcitability.

To further investigate the major neuronal subtypes in the dmPFC involved in mediating CMP, we examined changes in the activity of excitatory and inhibitory neurons in this region of CMP rats (Fig. 2u and Supplementary Fig. 2r). The results showed a notable increase in the number of cFos⁺ excitatory glutamatergic (VgluT2) neurons in the dmPFC, whereas the number of cFos⁺ inhibitory neurons (GAD65&67) did not show a statistically strong change (Fig. 2v and Supplementary Fig. 2s). Subsequently, in CMP (MP-28d) rats, we used optogenetics to activate CamKIIα-expressing excitatory glutamatergic neurons in the dmPFC (Fig. 2w). This activation notably increased the mechanical pain threshold (Fig. 2x). Moreover, in OFTs, the total distance traveled, as well as the percentage of time and distance spent in the center area, were markedly lower compared with the sham-activation group (Fig. 2y, z). Collectively, our findings demonstrated that the suppressed dmPFC, specifically the excitatory glutamatergic neurons, can mediate the generation and maintenance of CMP by inducing the hyperactivation of SDH.

### Reduced plasticity of excitatory synapse exists within the dmPFC in CMP

**Fig. 3:**
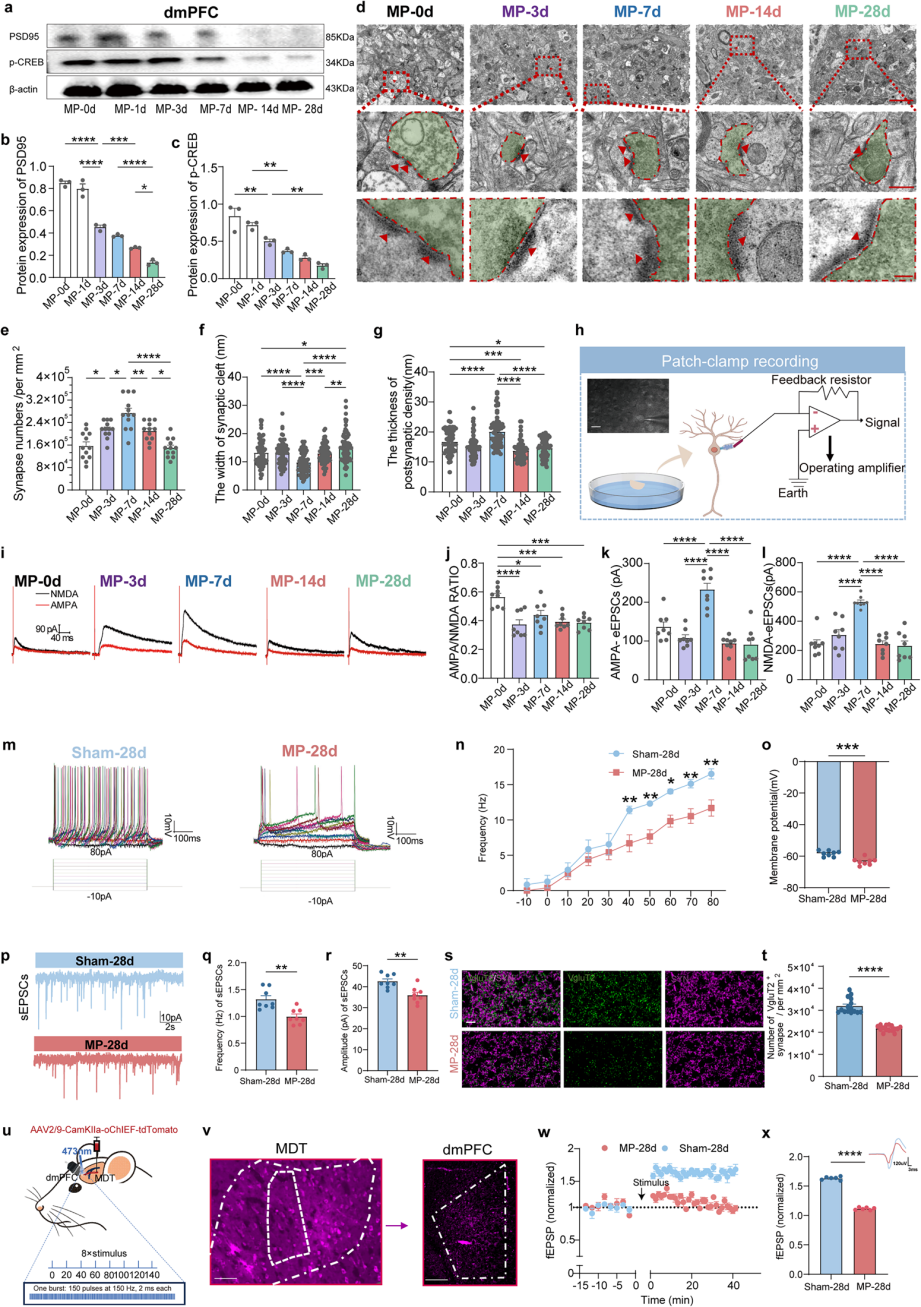
Reduced plasticity of excitatory synapse within dmPFC in CMP rats. **a** WB expression of PSD95 and pCREB within dmPFC. **b**, **c** The quantification of PSD95 (**b**) and pCREB (**c**) (*n* = 3 from six rats, two-tailed unpaired Student’s *t*-test). **d** The TEM images of the dmPFC. Scale bars, 2 µm and 0.5 µm. **e**–**g** The quantification of synapses via TEM (one-way ANOVA with Bonferroni’s post hoc test): the quantificaton of synapse numbers (*n* = 12 from six rats) (**e**) the quantification of the width of synaptic cleft (*n* = 72 from six rats) (**f**) and the quantification of the thickness of PSD (*n* = 63 from six rats) (**g**). **h** A schematic of whole-cell patch-clamp recordings from dmPFC neurons. Scale bar, 50 µm. **i** The representative traces of AMPA receptor- and NMDA receptor-mediated currents in baseline and MP-affected rats. **j**–**l** A summary of AMPA/NMDA EPSC ratios across groups from MP-0d, MP-3d, MP-7d, MP-14d to MP-28d (*n* = 8 from six animals, one-way ANOVA with Bonferroni’s post hoc test), and panel **j** shows the AMPA/NMDA eEPSC ratio, panel **k** shows AMPA-eEPSCs, and panel **l** shows NMDA-eEPSCs. **m** Voltage responses of pyramidal neurons to graded current injections, indicating APs. **n** Changes in action potential frequency. **o** Changes in action potential membrane potential (*n* = 8 from six animals, two-tailed unpaired Student’s *t*-test). **p** Typical traces of sEPSCs. **q**, **r** A summary of sEPSC frequency (**q**) and amplitude (**r**). **s** The IF images showing the colabeling of VgluT2 and SYN in dmPFC. Scale bar, 20 mm. **t** The quantification of VgluT2^+^ and SYN ^+^ synapse numbers (*n* = 6 rats, two-tailed unpaired Student’s *t*-test). **u** A schematic illustrating in vivo filed recording protocol. **v** A representative coronal section showing tdTomato^+^ axonal terminals projected from the MDT to dmPFC. Scale bar, 0.5 mm. **w** Averaged slopes of normalized fEPSPs before and after stimulus. **x** The quantification of fEPSPs. Statistical significance is exhibited as *P < 0.05; **P < 0.01; ***P < 0.001; ****P < 0.0001; ns, not significant.

It is considered that synaptic activity is fundamental for the neuronal excitability, and alterations in synaptic structure and function directly lead to the abnormal neuronal activity balance. Here, we conducted a comprehensive examination of synaptic architecture and functionality. First, we examined the protein levels related to synaptic plasticity using WB. The expression of PSD95 and pCREB in the dmPFC of CMP rats was reduced obviously compared with the control group (Fig. 3a–c). Using transmission electron microscopy (TEM) imaging, we quantified synapse counts in the dmPFC throughout the progression of CMP. We observed a remarkable increase in synapse numbers on day 3 and 7, followed by a gradual decrease at 14 and 28 days (Fig. 3d, e). We also examined the PSD, an electron-dense structure beneath the postsynaptic membrane^24^. TEM studies identified the PSD as a thickened electron-dense area below the plasma membrane of each excitatory synapse. The images showed that animals with CMP consistently exhibited increased synaptic gap width and reduced PSD at 14 and 28 days (Fig. 3f, g), indicating a structural remodeling of the synapses in CMP rats.

To delve deeper into synaptic functional plasticity, we performed patch-clamp experiments on dmPFC pyramidal neurons (Fig. 3h). In the context of CMP, we observed a notable decrease in the AMPA/NMDA ratio (Fig. 3i), and AMPA and NMDA eEPSCs decreased evidently on day 14 and 28 (Fig. 3j–l). To determine whether neurons were hyperactive or deactivated in CMP, we recorded action potentials of dmPFC pyramidal neurons on day 28 (Fig. 3m), where all neurons exhibited lower conductance, indicated by a nonlinear decrease in frequency (Fig. 3n) and amplitude (Fig. 3o). In our study, we measured spontaneous excitatory postsynaptic currents (sEPSCs) and spontaneous inhibitory postsynaptic currents (sIPSCs) in dmPFC pyramidal neurons using voltage-clamp recordings CMP rats. The results showed an obvious decrease in the frequency and membrane amplitude of sEPSCs (Fig. 3p–r). However, there are minimal changes in the frequency and amplitude of sIPSCs (Supplementary Fig. 3a, b). Given the changes in excitatory and inhibitory postsynaptic potentials, we further investigated the alterations in excitatory and inhibitory synapses in the dmPFC of CMP rats by the costaining of SYN/VgluT/GAD65&67 (Fig. 3s and Supplementary Fig. 3c). The results exhibited that excitatory synapse density was remarkably reduced (Fig. 3t), with no obvious change in inhibitory synapses (Supplementary Fig. 3d). To further compare the excitatory synaptic responses in the dmPFC, we applied an optical LTP (oLTP) protocol to photostimulated MDT–dmPFC axonal terminals consisting of eight high-frequency stimulation by in vivo field recording^25,26^. Using optogenetics, we activated excitatory synapses in the dmPFC arising from the MDT and recorded fEPSPs in the dmPFC (Fig. 3u, v). In the CMP group, this oLTP protocol produced a long-lasting decrease in fEPSP amplitude in the dmPFC (Fig. 3w, x). Together with the above results, the obviously aberrant remodeling of excitatory synaptic structure and function has been found in CMP rats.

### scRNA-seq links proinflammatory microglia to abnormal dmPFC plasticity in CMP

**Fig. 4:**
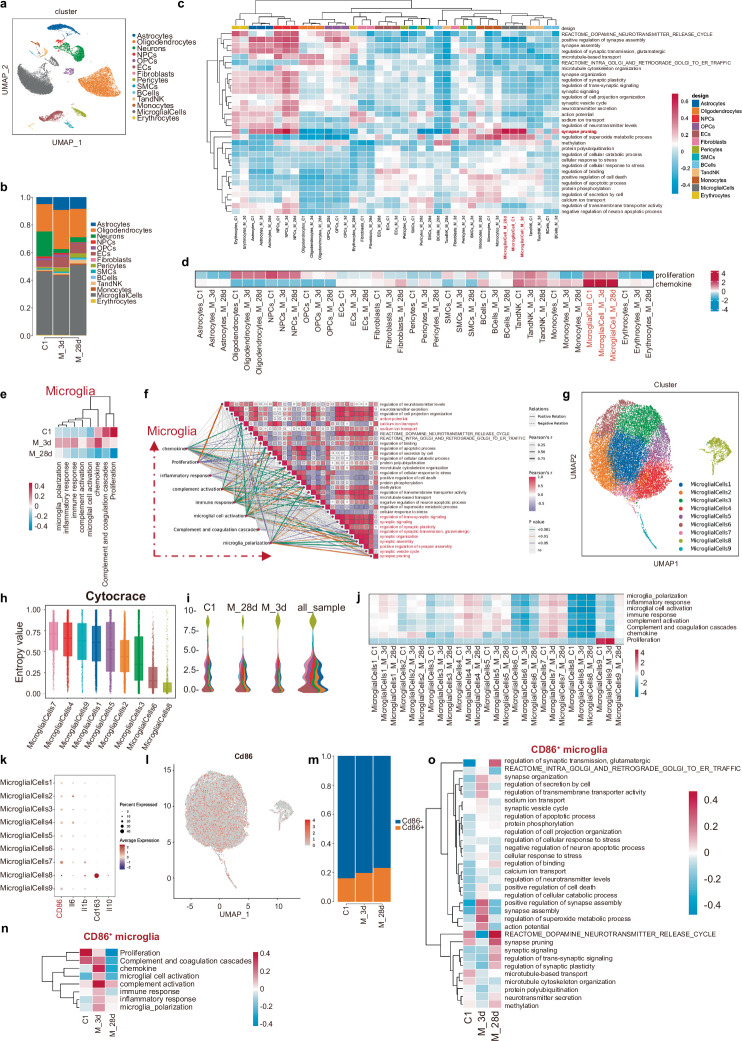
Proinflammatory microglia highly correlates with abnormal dmPFC plasticity in CMP. **a** A UMAP plot displaying the distribution of all cells in integrated dmPFC datasets, color-coded by cell type of Ctrl, MP-3d and MP-28d. **b** The stacked bar chart of dmPFC cell subset frequencies. **c** A heat map of enrichment scores for differentially enriched gene ontology terms related to neuronal activity and synaptic remodeling across all cell types among the three groups. **d** A heat map of enrichment scores for differentially enriched genes linked to proliferation and chemokines across the groups. **e** A heat map of enrichment scores for differentially enriched gene ontology terms associated with eight microglial functions among the groups. **f** The correlation analysis between microglial function, neuronal activity and synaptic remodeling; purple and red circles indicate positive and negative associations, respectively, with cube size representing the correlation coefficient. Solid and dotted lines denote positive and negative correlations. **g** A UMAP plot of microglia classified by mechanical clusters, colored by subtype. **h** The cytotrace analysis of all microglia subtypes. **i** A monocle analysis of microglia subtypes, with pseudotime on the vertical axis and cell type proportions over time on the horizontal axis. **j** A heat map of enrichment scores for gene ontology terms related to eight microglial functions across the three groups. **k** A dot plot showing expression levels of proinflammatory (CD86, Il6, Il1b) and anti-inflammatory (CD163, Il10) marker genes across microglia subtypes; dot color indicates normalized gene expression, and size reflects the percentage of cells expressing each gene. **l** A UMAP plot of CD86^+^ microglia. **m** A stacked bar chart of CD86^+^ microglia frequencies. **n** A heat map of enrichment scores for gene ontology terms related to eight microglial functions among CD86^+^ microglia across the groups. **o** A heat map of enrichment scores for gene ontology terms associated with neuronal activity and synaptic remodeling within CD86^+^ microglia across the groups.

To investigate the cellular and molecular mechanisms of CMP, we performed single-cell sequencing on dmPFC tissue. Quality control revealed 14 transcriptionally distinct cell types, with microglia being the most abundant (Fig. 4a, b and Supplementary Fig. 4a, b). We identified microglia as the critical cell type linked to neuronal activity and synaptic plasticity, showing the strongest correlation with synaptic pruning (Fig. 4c and Supplementary Fig. 4c, d). Ucell scores indicated strong associations between microglia and proliferation and chemokine expression during CMP (Fig. 4d and Supplementary Fig. 4e, f). Moreover, genes related to activated microglial functions were changed obviously in MP-affected animals, and these pathways correlated with neuronal activity and synaptic remodeling (Fig. 4e, f). These findings suggest that microglia in the dmPFC play a key role in the progression of CMP.

Next, we try to identify specific microglial subtypes. We found nine distinct subtypes through clustering analysis (Fig. 4g and Supplementary Fig. 4g–i). Cytotrace (Fig. 4h) and Monocle analyses (Fig. 4i and Supplementary Fig. 4j, k) indicated that cluster 8 remarkably differed from others, showing the lowest enrichment scores across all groups (Fig. 4j and Supplementary Fig. 5a, b). This led us to hypothesize that the remaining clusters, rather than cluster 8, are crucial in CMP. Microglial gene correlation identified eight functional modules, with all major clusters except cluster 8 linked to module 7, associated with synaptic pruning (Supplementary Fig. 5c–e). The characterization of the clusters based on inflammatory markers revealed that clusters 1–7 and 9 were CD86^+^, whereas cluster 8 was predominantly CD163^+^ (Fig. 4k), a pattern reflected in the uniform manifold approximation and projection (UMAP) plot (Fig. 4l and Supplementary Fig. 5f). The assessment of cell ratios showed an increased frequency of CD86^+^ microglia (proinflammatory subtype) (Fig. 4m) and a decrease in CD163^+^ microglia (anti-inflammatory subtype) (Supplementary Fig. 5g–i) after CMP. CD86^+^ microglia correlated strongly with activated functions, neuronal activity and synaptic remodeling (Fig. 4n, o), whereas CD163^+^ microglia showed minimal correlations (Supplementary Fig. 5j, k). Thus, we propose that CD86^+^ proinflammatory microglia might be the key cellular players responsible for suppressed dmPFC in CMP.

### Abnormal neuron- proinflammatory microglia interactions lead to reduced synaptic plasticity and suppressed dmPFC

**Fig. 5:**
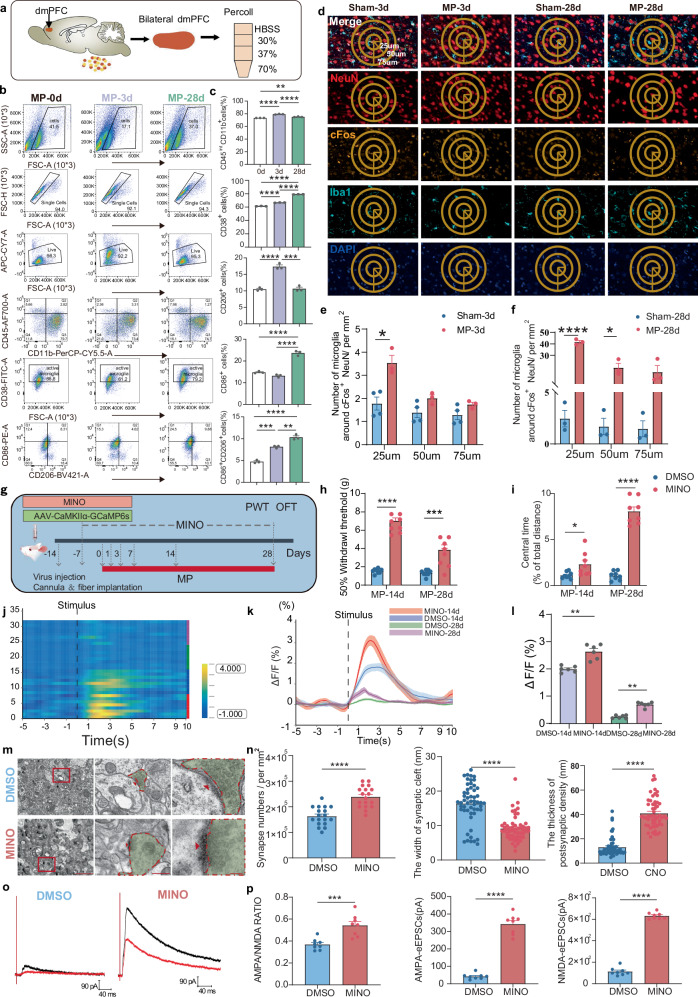
Proinflammatory microglia reduced synaptic plasticity and suppressed dmPFC in CMP. **a** A schematic for flow cytometry analysis. **b** The flow cytometry of dmPFC single-cell suspension, identifying microglia (CD45^int-^CD11b^+^), activated microglia (CD38^+^), proinflammatory microglia (CD86^+^ CD206^−^) and anti-inflammatory microglia (CD86^−^CD206^+^). **c** Population quantification histograms from up to down: microglia, activated microglia, anti-inflammatory microglia, double-positive microglia, proinflammatory microglia (*n* = 3 from 8 rats, one-way ANOVA with Bonferroni’s post hoc test). **d** IF images showing the colabeling of neurons (NeuN), neuronal activity (cFos), microglia (Iba1) and nuclei (DAPI) in dmPFC. Scale bars, low magnification 200 µm, high magnification 20 µm. **e**, **f** The quantification of microglia around cFos^+^ neurons at distances of 25 µm, 50 µm and 75 µm: Sham-3d and MP-3d (*n* = 4 rats in sham group, *n* = 3 rats in MP group) (**e**) MP-28d and Sham-28d (*n* = 3 rats, two-way ANOVA with Bonferroni’s post hoc test) (**f**). **g** The experimental scheme for MINO administration. PWT and OFT were conducted on day 28. **h** The PWT quantification following MINO administration. **i** The central time percentage (*n* = 8 rats, one-way ANOVA with Bonferroni’s post hoc test). **j** A heat map depicting dmPFC neuronal response to hind paw laser stimuli post-MINO on days 14 and 28 (eight trials per animal). The dashed white line indicates the stimulus timing. **k** Representative photometric traces. **l** The quantification of GCaMP6s signals in dmPFC glutamatergic neurons elicited by hind paw stimuli (*n* = 6 rats, 8 trials each, two-tailed Student’s *t*-test). **m** TEM images of dmPFC. Scale bars, 2 µm and 0.5 µm. **n** The synapse quantification from TEM (from six rats, one-way ANOVA with Bonferroni’s post hoc test). **o** Representative traces of AMPA and NMDA receptor-mediated currents in rats treated with MINO or DMSO. **p** A quantitative summary of AMPA/NMDA ratios for both genotypes (*n* = 8 from six rats, two-tailed Student’s *t*-test). Statistical significance is exhibited as *P < 0.05; **P < 0.01; ***P < 0.001; ****P < 0.0001; ns, not significant.

The flow cytometry of dmPFC microglia during CMP progression (Fig. 5a, b) revealed stage-specific polarization shifts. At 3 days post MP, we observed increased total microglia (CD45^int^CD11b^+^), activated (CD38^+^), anti-inflammatory (CD206^+^CD86^−^) and double-positive populations subsets, whereas proinflammatory (CD86^+^CD206^−^) decreased (Fig. 5c). This pattern reversed by day 7, followed by the substantial expansion of all subsets from days 14 to 28, suggesting biphasic microglial activation. Cytokine profiling matched these cellular changes: early phase (3 days) showed upregulated proinflammatory markers (IL1β, TNF-α, IL6, iNOS), whereas late phase (28 days) demonstrated anti-inflammatory dominance (IL4, IL10, Arg1, BDNF) (Supplementary Fig. 6a, b). A spatial analysis revealed enhanced microglial recruitment to activated neurons (cFos^+^NeuN^+^) at 25–75 μm distances during both acute and chronic phases (Fig. 5d–f). The optogenetic activation of glutamatergic neurons confirmed this neuron–microglia crosstalk, inducing localized microglial accumulation (Supplementary Fig. 6c–f), with evident proximity effects at 25–75 μm radii and activation around stimulated neurons (cFos^+^). These findings demonstrate activity-dependent microglial recruitment to hyperactive neuronal circuits during CMP progression.

To confirm the critical role of microglia in modulating the inhibited neural activity of the dmPFC, we suppressed microglia through the intracerebroventricular (ICV) administration of MINO in rats 1 week before the onset of CMP (Fig. 5g). After 28 days, a flow cytometry analysis revealed an obvious significant reduction in the percentage of both microglia and activated microglia (Supplementary Fig. 6g–j). Simultaneously, a decrease in pain behaviors was noted (Fig. 5h, i and Supplementary Fig. 6k–m). Furthermore, following the MINO treatment, the dmPFC neurons demonstrated pronounced activation in response to nociceptive stimuli applied to the ipsilateral hind paw 28 days post MP, as indicated by the 473-nm wavelength (Fig. 5j–l). The IF results corroborated this trend, showing reduced microglial presence around activated neurons in the dmPFC (Supplementary Fig. 6n).

Notably, the previously observed structural alterations were reversed following MINO administration (Fig. 5m, n). Specifically, there was an increase in synapse numbers, a thickening of the PSD and a reduction in the synaptic gap within the dmPFC, even suffering from CMP. These results emphasize the essential role of microglia in mediating synaptic structural remodeling in the dmPFC during the CMP. Furthermore, the microglia inhibition in MP rats was associated with an elevated AMPA/NMDA ratio, suggesting that microglia may contribute to synaptic depression (Fig. 5o, p). Thus, these findings suggest that the activated proinflammtory microglia may act as crucial regulators for suppressed dmPFC and reduced synaptic plasticity associated with CMP.

### Microglial CR3 expression correlates with dmPFC excitability and synaptic plasticity

**Fig. 6:**
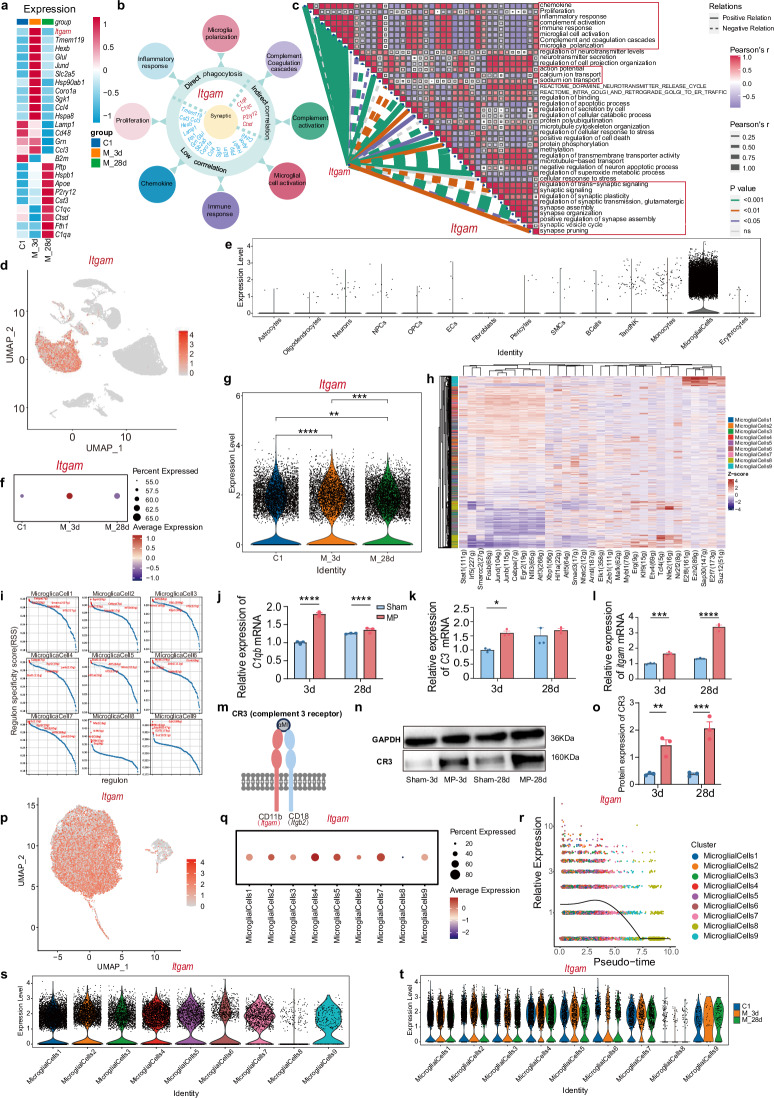
Microglial CR3 is highly correlated with the excitability and synaptic plasticity of dmPFC. **a** A heat map displaying notable genes related to eight activated microglial function pathways. **b** Images categorizing the top 22 genes into three groups on the basis of synapse engulfment. **c** A correlation analysis between itgam and pathways associated with neuronal activity and synaptic remodeling. **d** A UMAP plot of *itgam*^+^ cells from integrated dmPFC datasets across all cell types. **e** A box plot comparing enrichment scores of *itgam* among all cell types. **f** A dot plot illustrating itgam expression in CD86^+^ microglia across three groups. **g** A violin plot of *itgam* expression across three groups. **h** A heat map of key upstream transcription factors. **i** The regulon specificity score for the top upstream transcription factor. **j**–**l** The quantification of *C1qb* mRNA expression (**j**) *C3* mRNA expression (**k**) and *itgam* mRNA expression (**l**) (*n* = 3 from six rats, two-tailed paired or unpaired Student’s *t*-test). **m** A schematic representation of CR3 structure, including CD11b (encoded by *itgam*) and CD18 (encoded by *itgb*2). **n** Representative WB images of CR3 in dmPFC. **o** The quantification of CR3 protein expression (*n* = 3 from six rats, two-tailed paired or unpaired Student’s *t*-test). **p** A UMAP plot of itgam^+^ cells in nine microglial subtypes. **q** A dot plot showing itgam expression across nine microglial subtypes. **r** A pseudotime analysis of itgam expression within nine microglial subtypes. **s** A violin plot of itgam expression among nine microglial subtypes. **t** A violin plot of itgam expression across nine microglial subtypes in three groups. Statistical significance is exhibited as ***P < 0.001; ****P < 0.0001; ns, not significant.

The above studies identified proinflammatory microglia in the dmPFC as key contributors to CMP. To investigate the underlying molecular mechanisms, we conducted a bioinformatic analysis to identify key genes. By comparing enrichment scores among three groups in a dataset of eight microglial function-related pathways, we identified 25 genes with relative expression levels above 4.5 (Fig. 6a). The correlation analysis revealed that 22 of these genes were markedly associated with neuronal activity and synaptic plasticity pathways, whereas Jund, Hsp90ab1 and Hspa8 showed no such association (Supplementary Fig. 7a). Notably, Itgam (encoding CR3) exhibited the strongest correlation with synaptic pruning in CMP versus controls (Fig. 6b), high association with neuronal activity and synaptic plasticity (Fig. 6c) and specific expression in microglia (Fig. 6d, e).

In our study, *itgam* was evidently upregulated following MP modeling, particularly 3 days later (Fig. 6f, g). This upregulation was supported by the increased expression of upstream transcription factors, including Cebpa, Fosb, Junb and Jund (Fig. 6h, i). We further examined *itgam* and its upstream genes (*C1qb, C3*) associated with the classical complement pathway, finding remarkable increases in their expression (Fig. 6j–l). Western blotting (WB) analysis confirmed the marked upregulation of the itgam-encoded protein CR3 on day 3 and 28 suffering from MP (Fig. 6m–o). In addition, we assessed itgam distribution across nine microglial subtypes, revealing its increased expression in all subtypes except cluster 8 (Fig. 6p–s), particularly on day 3 (Fig. 6t), indicating its predominant presence in CD86^+^ proinflammatory microglia. These findings establish microglial CR3 as a critical regulator of neuronal excitability and synaptic plasticity in CMP pathogenesis.

### Microglial CR3 knockdown alleviates CMP by restoring excitatory synapses and enhancing glutamatergic activity

**Fig. 7:**
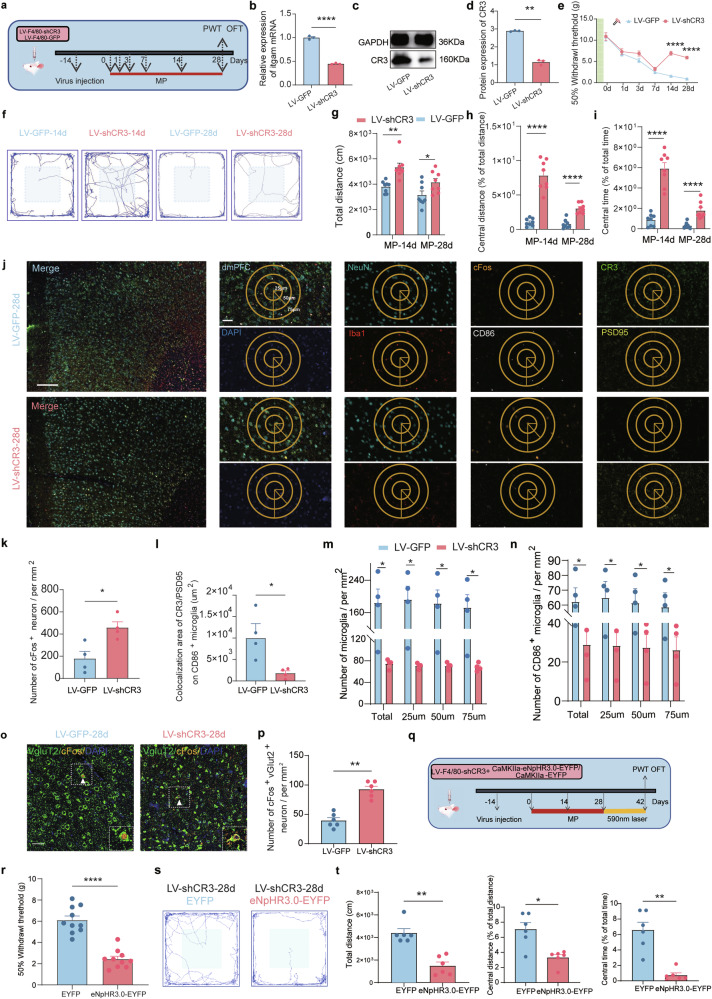
Microglial CR3 knockdown alleviates CMP by restoring glutamatergic activity. **a** An experimental scheme for CR3 (itgam) knockdown via lentivirus; PWT and OFT were assessed on day 28. **b** The relative expression of *itgam* post-knockdown (*n* = 3 from six rats, two-tailed unpaired Student’s *t*-test). **c** Representative WB images of CR3 in the dmPFC after transfection. **d** The quantification of CR3 protein levels (*n* = 3 from six rats, two-tailed unpaired Student’s *t*-test). **e** The PWT following CR3 knockdown (*n* = 10; two-way ANOVA with Bonferroni’s post hoc test. **f** The representative travel trace of OFT behaviors on day 14 and 28. **g**–**i** The quantification on day 14 and 28 (*n* = 8, two-tailed unpaired Student’s *t*-test) of total distance (**g**), central distance (**h**) and central time (**i**). **j** The mIHC in dmPFC after knockdown (scale bars, low magnification 100 µm, high magnification 50 µm). **k** The cFos^+^ neuron quantification. **l** The colabeling of CR3 and PSD95 in CD86^+^ microglia. **m** The microglia density per µm^2^: the total microglia and microglia counts at distances of 25 µm, 50 µm and 75 µm from cFos^+^ neurons per µm^2^ from left to right. **n** The CD86^+^ microglia density per µm^2^: the total CD86^+^ microglia and their counts at distances of 25 µm, 50 µm and 75 µm from cFos^+^ neurons per µm^2^. In **k**–**n** *n* = 4, two-tailed unpaired Student’s *t*-test. **o** The IF colocalized of vGluT2 with cFos in dmPFC after knockdown CR3 (scale bars, 50 µm). **p** The number of cFos^+^ vGluT2^+^ neuron in dmPFC after knockdown CR3 (*n* = 6, two-tailed unpaired Student’s *t*-test). **q** The experimental scheme for CR3 (itgam) knockdown and optogenetic inhibition of CamKIIa neurons. **r** PWT following CR3 knockdown in CMP rats and accepted activation of glutamatergic neurons for 2 weeks (*n* = 10; two-way ANOVA with Bonferroni’s post hoc test. **s** A representative travel trace of OFT. **t** The quantification of traveling following CR3 knockdown followed by optogenetic activation (*n* = 6, two-tailed unpaired Student’s *t*-test). Statistical significance is exhibited as *P < 0.05; **P < 0.01; ***P < 0.001; ****P < 0.0001; ns, not significant.

To elucidate the role of microglial CR3, we knocked down its expression (Fig. 7a). PCR and WB confirmed the successful knockdown (Fig. 7b–d). Mechanical pain was relieved in injured hind limbs (Fig. 7e), and OFT showed increased exploration, indicating reduced anxiety-like behaviors on day 14 and 28 (Fig. 7f–i), whereas these changes were not found on day 3 (Supplementary Fig. 8a–d). The IF revealed more activated neurons (Fig. 7j, k), whereas the colabeling of Iba1/PSD95/CR3 decreased, suggesting reduced synaptic phagocytosis (Fig. 7l). Microglia numbers decreased (Fig. 7m), as did CD86^+^ proinflammatory microglia near activated neurons (Fig. 7n). The flow cytometry confirmed decreased microglial populations at day 28 post knockdown (Supplementary Fig. 8e, f), including activated microglia (Supplementary Fig. 8g), CD86^+^ subsets (Supplementary Fig. 8h, i) and increased CD206^+^ anti-inflammatory microglia (Supplementary Fig. 8j). The MINO administration inhibited itgam increasing (Supplementary Fig. 8k). Moreover, the colabeling of vGluT2 and cFos increased after microglial CR3 knockdown (Fig. 7o, p), whereas there is no obvious difference of GAD65&67 and cFos labeling (Supplementary Fig. 8l, m).

In the MP-28d rat model group, the knockdown of microglial CR3 followed by continuous optogenetic inhibition of excitatory glutamatergic neurons for 14 days resulted in a decreased mechanical pain threshold (Fig. 7q, r) and a markedly increase in anxiety-like behaviors in the OFT (Fig. 7s, t). The above results indicate that during chronic pain processing, microglial CR3 suppresses the activity of excitatory glutamatergic neurons in the dmPFC, thereby promoting the development of CMP.

**Fig. 8:**
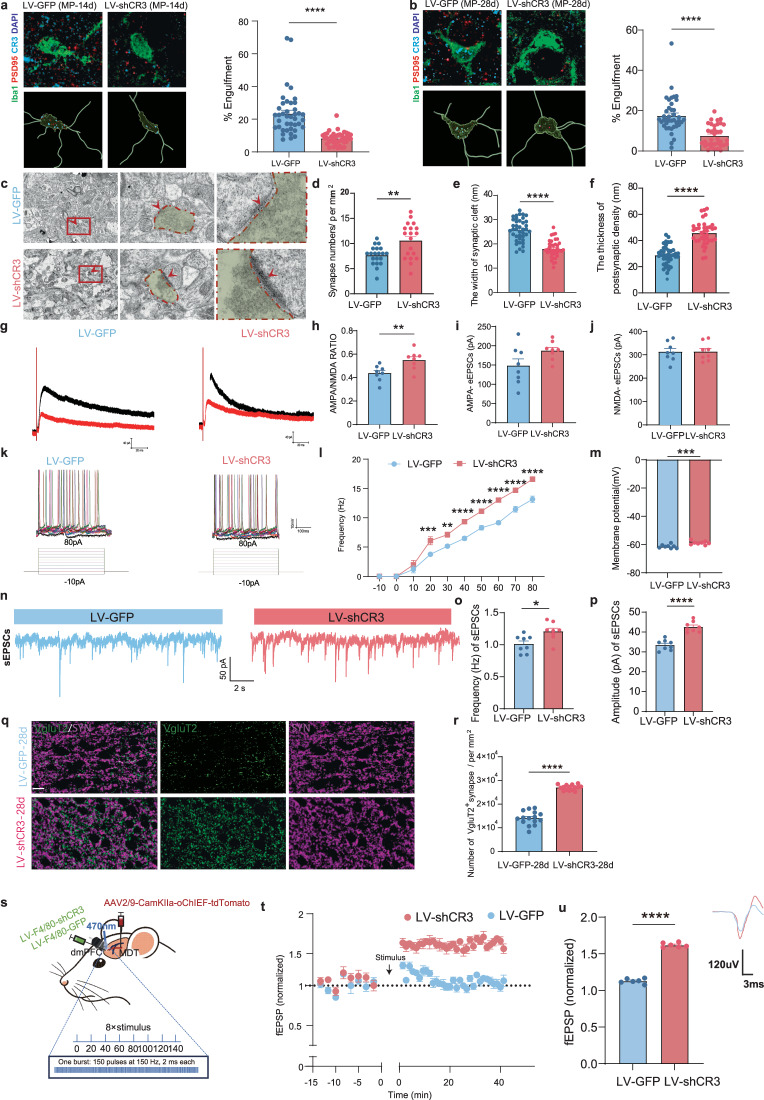
Microglia CR3 knockdown ameliorates synaptic impairment in CMP. **a**, **b** A confocal 3D reconstruction of Iba1, PSD95, CR3 and DAPI costaining and quantification of microglial synapse engulfment percentage for 14 days (a) and 28 days (b) after MP accepting CR3 knockdown group or GFP control group (*n* = 35–40 randomly sampled microglial cells per animal from four independent animals, two-tailed paired or unpaired Student’s *t*-test). **c** The TEM images of dmPFC post-CR3 knockdown (scale bars, 2 µm and 0.5 µm). **d**–**f** The quantification of synapses from TEM: synapse number (**d**), the width of the synaptic cleft (**e**) and the thickness of PSD (**f**) (from six rats, two-tailed unpaired Student’s *t*-test). **g** Representative traces for AMPA and NMDA receptor-mediated currents after CR3 knockdown. **h**–**j** A summary of AMPA/NMDA EPSC ratios: AMPA/NMDA ratio (**h**) eEPSCs of AMPA (pA) (**i**) and eEPSCs of NMDA (pA) (**j**) (*n* = 8 from six animals, two-tailed unpaired Student’s *t*-test). **k** The voltage responses of pyramidal neurons to graded current injections. Left, LV-GFP. Right, CR3 knockdown. **l** Changes in action potential frequency. **m** The changes in action potential membrane potential (*n* = 8 from six animals, two-tailed unpaired Student’s *t*-test). **n** Typical traces of sEPSCs. **o**, **p** A summary of sEPSC frequency (**o**) and amplitude (**p**). **q** The IF images showing the colabeling of VgluT2 and SYN in dmPFC. Scale bar, 20 mm. **r** The quantification of VgluT2^+^ and SYN ^+^ synapse numbers (*n* = 6 rats, two-tailed unpaired Student’s *t*-test). **s** A schematic illustrating in vivo filed recording protocol. **t** Averaged slopes of normalized fEPSPs before and after stimulus. **u** The quantification of fEPSPs. Statistical significance is exhibited as *P < 0.05; **P < 0.01; ****P < 0.0001; ns, not significant.

We next investigated the relationship between microglial CR3 and aberrant synaptic remodeling. Multiplex IF revealed enhanced synaptic phagocytosis in MP-28d versus sham (Supplementary Fig. 9a), which CR3 knockdown attenuated (Fig. 8a, b). Reduced Iba1/PSD95/CR3 colocalization in LV-shCR3-28d rats further confirmed diminished synaptic pruning. An ultrastructural analysis showed CR3 knockdown increased synaptic density, thickened PSDs and narrowed synaptic clefts (Fig. 8c–f). Electrophysiological recordings demonstrated improved AMPA/NMDA ratios (Fig. 8g–j) and elevated pyramidal neuron excitability (increased firing frequency (Fig. 8k, l) and membrane nonlinearity (Fig. 8k, m)). Moreover, recordings from dmPFC pyramidal neurons showed an evident increase in sEPSC frequency and amplitude (Fig. 8n–p), whereas sIPSCs displayed no big difference (Supplementary Fig. 9b, c). We also found the increased colabeling of vGluT2 and SYN after microglial CR3 knockdown (Fig. 8q, r), whereas there is no evident difference of colabeling of GAD65&67 and SYN (Supplementary Fig. 9d, e). The further oLTP protocol produced a long-lasting increase in fEPSP amplitude in the dmPFC, following microglial CR3 knockdown (Fig. 8s–u). These results indicate that the microglial CR3 knockdown alleviates CMP by restoring the excitability and synaptic plasticity of glutamatergic neurons in the dmPFC.

## Discussion

In this study, we first provide compelling evidence that suppressed glutamatergic neuronal excitability and reduced synaptic plasticity within dmPFC mediated by microglial CR3 synaptic pruning promote the generation and maintenance of CMP. Using a combination of fiber photometry, electrophysiological recording and optogenetic and chemogenetic manipulations, we demonstrated that activating the dmPFC excitatory neuron activity alleviates both pain and anxiety-like behaviors in CMP model rats. Importantly, our scRNA-seq results revealed a pronounced upregulation of microglial CR3, linking proinflammatory microglial activation to glutamatergic dysfunction in the dmPFC. Notably, the inhibition of microglia or knockdown of microglial CR3 restored dmPFC glutamatergic neuronal excitability and synaptic plasticity, thereby alleviating mechanical hyperalgesia and anxiety-like behaviors of CMP rats. These findings advance CMP pathophysiology by highlighting a key neuron–microglia interaction mediated through CR3-dependent synaptic pruning that underlies both pain hypersensitivity and emotional comorbidities.

Although reduced dmPFC activity is documented in chronic pain^27,28^, we uncover dynamic phase-dependent modulation: initial excitation shifts to inhibition during chronicity, aligning with prior models^6,8^. Chemogenetic dmPFC activation alleviated pain and anxiety in CMP rats, whereas early phase inhibition exacerbated symptoms, corroborating the PFC’s antinociceptive role^29,30^. Paradoxically, the optogenetic suppression of dmPFC outputs enhanced pain control^31^, highlighting its context-dependent regulatory function in nociception. We further explored an inverse dmPFC–SDH activity relationship, indicating dmPFC-mediated SDH suppression, as dmPFC–vlPAG pathways regulate descending pain control^32–34^. We demonstrate that dmPFC inhibition disrupts this modulation, inducing SDH hyperactivation and CMP. We identify a key role for dmPFC glutamatergic neurons in CMP. Despite the overall dmPFC suppression, cFos⁺ excitatory neurons showed increased activity—suggesting a maladaptive plasticity during persistent pain^35^. Inhibitory neurons remained unchanged, implicating excitatory circuits specifically in CMP^36^. The optogenetic activation of CamKIIα⁺ neurons alleviated mechanical hypersensitivity^37^ but reduced exploratory behavior (OFT), linking dmPFC excitation to both analgesia and anxiety-like states. These findings demonstrate that suppressed dmPFC glutamatergic excitability drives the generation and maintenance of CMP.

We observed progressive synaptic deficits in dmPFC during CMP progression, including synapse loss, thinner PSDs and widened synaptic clefts—structural changes that impair excitatory transmission and neuronal excitability. These alterations emerged between days 7–28 post injury, coinciding with dmPFC functional suppression and sustained pain sensitization from day 14 onward^38^. The temporal alignment of synaptic degeneration with chronic-phase behavioral and physiological changes suggests these structural modifications drive dmPFC hypoactivity and serve as critical transition markers in CMP pathogenesis. Patch-clamp recordings revealed dmPFC synaptic dysfunction in CMP, featuring reduced AMPA/NMDA ratios, sEPSCs and action potential firing—consistent with pyramidal neuron hypoactivity—indicate synaptic disinhibition. In parallel, glutamatergic synapses are markedly decreased, and LTP is impaired, whereas inhibitory synapse counts remain stable, resulting in reduced excitatory input to the dmPFC^8^. The overall inhibitory activity appears unchanged, where the relative proportions of inhibitory interneuron subtypes (PV, SST, VIP) may shift^11^.

These findings demonstrate excitation–inhibition imbalance via glutamatergic synapse loss—not postsynaptic receptor changes—aligning with chronic pain synaptic pathology^35,39–43^. Together, our findings demonstrate that excitatory synapses within the suppressed dmPFC undergo reduced plasticity in CMP rats.

Our study establishes microglia as pivotal regulators of dmPFC hypoexcitability in CMP, where scRNA-seq revealed CR3-mediated microglial signatures correlating with synaptic pruning and neuronal activity pathways. We observed that early microglial proliferation and proinflammatory polarization (peaking at days 14–28) create a maladaptive feedback loop: neuronal activation drives microglial recruitment (25–75 μm proximity)^44,45^. We establish microglial CR3 as a central regulator of CMP, where its upregulation during chronic phases (days 14–28) drives synaptic dysfunction via enhanced proinflammatory microglial activation and suppresses glutamatergic neuron activity. The CR3 knockdown attenuated pain behaviors, demonstrating its pathogenic role in inflammatory signaling. These findings establish CR3 as both a biomarker and therapeutic target for chronic pain progression. We demonstrate that microglial CR3 critically regulates neuronal excitability in CMP, where CR3 knockdown reduced proinflammatory microglial activation near glutamatergic neurons while enhancing their activity. Both genetic and pharmacological (MINO) CR3 suppression attenuated microglial chemotaxis and inflammation, with behavioral protection contingent on maintained excitatory neuron activity—as optogenetic inhibition reversed analgesic effects^26,46^. Although complement-mediated pruning maintains neural circuits physiologically, CR3-dependent dysregulation drives pathological glutamatergic synaptic loss and disability in chronic pain. We demonstrate that microglial CR3 knockdown alleviates CMP by restoring glutamatergic excitability and synaptic plasticity in the dmPFC^12,46–48^.

In conclusion, our study demonstrates that microglial CR3-dependent synaptic pruning in the dmPFC suppresses glutamatergic neuronal excitability and synaptic plasticity, contributing to the generation and maintenance of CMP and its associated anxiety-like behaviors. These findings first highlight the key microglia–neuron interaction for the generation and, especially, the maintenance of CMP and provide new intervention targets for effectively preventing its recurrence. To advance this work, future studies should address two key gaps: (1) the unresolved contribution of inhibitory neurons and synapses to CMP and (2) whether microglia preferentially engulf long-range or intracortical excitatory synapses.

## Supplementary information

Supplementary Information

Supplementary Table 1
