# Supplementary material for: Microglial CR3-mediated synaptic pruning in the dmPFC promotes the generation and maintenance of chronic muscle pain via glutamatergic dysfunction
Source: Exp Mol Med. 2026 Mar 2;58(3):664–80. doi: 10.1038/s12276-026-01666-7 (PMC13049173; doi:10.1038/s12276-026-01666-7)
Supplement: Supplementary file 2 — Supplementary Table 1 [file 12276_2026_1666_MOESM2_ESM.pdf]

Supplementary Table 1. qPCR Supplemental file

**Gene name**	**Forward 5′-……-3′**	**Reverse 5′-……-3′**
C1qb	TCAACAGCGCCCTGCGACCAAACCA	TGAACTTGCCACTGCGCGGCTCGTA
C3	GCCAGCAGCTCTACAATGTG	GACTGCCACTTTCCCATAGC
itgam	AAACCCGAGTGGTTGTTGCAGCCCC	ATGGGGTCGCACCGGTTTGTGCTGT
BDNF	TGTGGTCAGTGGCTGGCTCTC	ACAGAACAGAACAGGACGGAAACAG
IL-10	ACTGCTATGTTGCCTGCTCTTACTG	GGTCTGGCTGACTGGGAAG
TNF-α	AAAGGACACCATGAGCACGGAAAG	CGCCACGAGCAGGAATGAGAAG
Arg-1	AGTGTGGTGCTGGGTGGAGAC	AGCGGAGTGTTGATGTCAGTGTG
CD206	TGGACAGACGGACGAGGAGTTC	GCCACCAATCACAACAACACAGTC
CD16	CAAAGTTCCGTGGCAGTCTATGAGG	GCAGATGGTGAGGTCGCAAGTC
iNOS	GAGACGCACAGGCAGAGGTTG	AGCAGGCACACGCAATGATGG
IL-1β	AATCTCACAGCAGCATCTCGACAAG	TCCACGGGCAAGACATAGGTAGC
IL-6	ACTTCCAGCCAGTTGCCTTCTTG	TGGTCTGTTGTGGGTGGTATCCTC
IL-4	CAAGGAACACCACGGAGAACGAG	TTCTTCAAGCACGGAGGTACATCAC
