# Supplementary material for: Microglial CR3-mediated synaptic pruning in the dmPFC promotes the generation and maintenance of chronic muscle pain via glutamatergic dysfunction
Source: Exp Mol Med. 2026 Mar 2;58(3):664–80. doi: 10.1038/s12276-026-01666-7 (PMC13049173; doi:10.1038/s12276-026-01666-7)
Supplement: Supplementary file 1 — Supplementary Information [file 12276_2026_1666_MOESM1_ESM.docx]

**Supplemental materials**

**Supplementary Fig.1. Thermal pain test and animal fMRI.** (a) Cartoon showing thermal pain test. (b) Mechanical thresholds of contralateral and ipsilateral hind paws post MP or sham stimulation (n = 8-10; two-way ANOVA with Bonferroni’s post hoc test). (c) Representative fMRI images showed in dmPFC according to Reho value (n =4, two-tailed paired or unpaired Student’s t-test). (d) and (e) Net Ca^2+^ flux changes, with (d) showing an 8-minute fluctuation and (e) total changes (n =6, one-way ANOVA with Bonferroni’s post hoc test). Statistical significance is exhibited as **P <0.05; **P<0.01; ***P<0.001; ****P<0.0001.*

**Supplementary Fig.2. Changes in SDH and vlPAG in CMP.** (a) Images showing the successful transcription of DREADDs virus. (b) IF images of the SDH. Scale bar: 100 µm. (c) Quantification of SDH IF results (n = 6, one-way ANOVA with Bonferroni’s post hoc test). (d) WB expression of PSD95 and p-CREB within SDH. (e) and (f) Quantification of PSD95 (left) and p-CREB (right) (n = 3 from 6 rats, two-tailed unpaired Student’s t-test). (g) Schematic of dmPFC and SDH projections. (h) IF images of cFos in SDH. Scale bar: 100 µm. (i) Quantification of cFos in SDH (n =6, two-tailed unpaired Student’s t-test). (j) Left: Schematic of anterograde tracing of dmPFC projection neurons using BDA and retrograde tracing of vlPAG neurons using FG; right: IF images of BDA (red) and FG (gold) tracers. Purple: DAPI. Scale bar: dmPFC 500 µm and 50 µm (insets); SDH 500 µm and 50 µm (insets). (k) NMT recordings in the dmPFC and SDH 28 days post-MP (n =6, two-tailed unpaired Student’s t-test). (l) NMT recordings in the vlPAG after DREADDs regulation (n =6, two-tailed unpaired Student’s t-test). (m) WB analysis of PSD95 and p-CREB of vlPAG in response to dmPFC activation. (n) Quantification of (m) (n= 3 from 6 rats, two-tailed unpaired Student’s t-test). (o) WB results following dmPFC inhibition. (p) Quantification of (o) (n= 3 from 6 rats, two-tailed unpaired Student’s t-test). (q) Schematic of dmPFC, vlPAG, and SDH projections. (r) The IF images showing co-label of GAD65&67 and cFos in dmPFC. Scale bar: 50 mm. (s) The quantification of GAD65&67^+^ and cFos^+^ neuron numbers (n=6 rats, two-tailed unpaired Student’s t-test). Statistical significance is exhibited as **P <0.05; **P<0.01; ***P<0.001; ****P<0.0001; ns, not significant.*

**Supplementary Fig.3. Changes of inhibitory neurons and IPSCs.** (a) Typical traces of sIPSCs. (b) Quantification of sIPSC frequency and amplitude (n = 8 from 6 animals, two-tailed unpaired Student’s t test). (c) The IF images showing co-label of GAD65&67 and SYN in dmPFC. Scale bar: 20 mm. (d) The quantification of GAD65&67^+^ and SYN^+^ synapse numbers (n = 6 rats, two-tailed unpaired Student’s t-test).

**Supplementary Fig.4. Subtype changes of microglia.** (a) Dot plot of top different gene of all cell subset in dmPFC. Dot size represents the percentage of cells that express a transcript, and color intensity represents the expression level of a transcript. (b) UMAP plot of all cells from integrated dmPFC datasets colored by cell type for 3 groups. (c) Heatmap depicting enrichment scores of differentially enriched gene ontology terms associated with neuronal activity and synaptic remodeling in dmPFC compared among 3 groups. (d) Heatmap depicting enrichment scores of differentially enriched gene ontology terms associated with neuronal activity and synaptic remodeling across all types of cells in dmPFC. (e) Left: box plot of enrichment scores of proliferation datasets compared among all sunsets, right: further comparison across 3 groups. (f) Left: box plot of enrichment scores of chemokine datasets compared among all sunsets, right: further comparison across 3 groups. (g-i) UMAP plot of microglia from integrated dmPFC datasets colored by cell type for 3 groups. (j) Monocle analysis of all microglia subtypes. The x-axis represents pseudotime, and the y-axis shows the density of cell counts at different time points (presenting cells analyzed across all parameters). Different colors indicate the density distribution of different cell types as they change over pseudotime. (k) A plot showing the pseudotime ordering for microglia; the sequence of time indicates the progression of pseudotime during differentiation.

**Supplementary Fig.5. Changes and possible functions of CD163^+^ microglia.** (a) Heatmap depicting enrichment scores of differentially enriched gene ontology terms associated with neuronal activity and synaptic remodeling functions in dmPFC compared among 9 microglia subtypes. (b) Heatmap depicting enrichment scores of differentially enriched gene ontology terms associated with 8 microglial functions in dmPFC compared among 9 microglia subtypes. (c) Heatmap of microglial gene correlations, with the annotations on the left representing gene sets. (d) UMAP plot of microglia gene set scores. (e) Dot plot of Module 7. The x-axis represents GeneRatio, and the y-axis represents GO Terms. The color of the circles indicates the p.adjust value, while the size of the circles represents the number of differentially expressed genes. (f) UMAP plot of CD163^+^ microglia. (g) Stacked bar chart of CD163^+^ frequencies in microglia among 3 group. (h) Stacked bar chart of CD163^+^ frequencies in 9 microglia subset. (i) UMAP plot of CD163^+^ microglia in 9 subsets. (j) Heatmap depicting enrichment scores of differentially enriched gene ontology terms associated with 8 microglial functions in dmPFC compared among 3 groups within CD163^+^ microglia. (k) Heatmap depicting enrichment scores of differentially enriched gene ontology terms associated with neuronal activity and synaptic remodeling in dmPFC compared among 3 groups within CD163^+^ microglia.

**Supplementary Fig.6. Flow cytometry and PCR results.** (a-b) Relative expression levels of 10 inflammatory cytokines in the dmPFC. (a) 3 days after surgery, (b) 28 days after surgery. (n= 3 rats, one-way ANOVA with Bonferroni’s post hoc test). (c) IF images showing co-labeling of NeuN/cFos/Iba1, M1 microglia (CD86), and nuclei (DAPI) scale bars: low magnification 50 µm. (d) Quantification of total number of microglia around cFos^+^ neurons. (e) Quantification of microglia around cFos^+^ neurons at distances of 25 µm, 50 µm, and 75 µm. (f) Quantification of total number of CD86^+^ microglia around cFos^+^ neurons (n=3, two-tailed unpaired Student’s t test). (g) dmPFC flow cytometry of DMSO and MINO groups. (h-j) Population quantification histograms from left to right: microglia, activated microglia, anti-inflammatory microglia, double-positive microglia, pro-inflammatory microglia (n= 3 from 24 rats, one-way ANOVA with Bonferroni’s post hoc test). (k) Representative travel trace of OFT behaviors on day 14 and 28. (l)) Total distance quantification. (m) Central distance percentage (n= 8 rats, one-way ANOVA with Bonferroni’s post hoc test). (n) Co-staining of dmPFC after MINO administration, scale bar: 50 µm. Statistical significance is exhibited as **P <0.05; **P<0.01; ***P<0.001; ns, not significant*.

**Supplementary Fig.7. Results of 25 Top genes correlation.** (a) Correlation between 25 top genes and neuronal activity and synaptic remodeling. Purple and red circles respectively indicate positive and negative association, and the size of each cube corresponds to the correlation coefficient. Solid line and dotted lines respectively indicate positive and negative correlation between microglial function, neuronal activity and synaptic remodeling.

**Supplementary Fig.8. Knockdown of Microglial CR3.** (a) Representative travel trace of OFT behaviors on day 3; (b-d) Quantification of traveling results in OFT (n = 8, two-tailed paired or unpaired Student’s t-test). (e) dmPFC flow cytometry of LV-GFP and LV-shCR3 groups. Top: Representative gate of LV-GFP group. Bottom: Representative gate of LV-shCR3 group. (f-j) Quantification of microglia after CR3 knockdown, (f) Quantification of microglia numbers, (g) Quantification of activated microglia numbers, (h) Quantification of CD86^+^ proinflammatory microglia. (i) Quantification of CD86^+^CD206^+^ microglia. (j) Quantification of CD86^-^CD206^+^ anti-inflammatory microglia. (f-j) (n = 8, two-tailed paired or unpaired Student’s t-test). (k) Quantification of relative expression of *itgam* after MINO administration (n = 3 from 6 rats, two-tailed paired or unpaired Student’s t-test). (l) IF co-localized of GAD65&67 with cFos in dmPFC after knockdown CR3 (scale bars: 50 µm). (m) Number of cFos^+^ GAD65+67^+^ neuron in dmPFC after knockdown CR3 (n = 6, two-tailed unpaired Student’s t-test). Statistical significance is exhibited as **P <0.05; **P<0.01; ***P<0.001; ****P<0.0001; ns, not significant*.

**Supplementary Fig.9. Synaptic plasticity after Microglial CR3 knockdown.** (a) Confocal reconstructed 3D images of Iba1, PSD95, CR3 and DAPI co-staining 28 days after MP, scale bar: 1µm. (m) Quantification of (l) (n= 35–40 randomly sampled microglial cells per animal from four independent animals). (b) Typical traces of sIPSCs. (c) Quantification of sIPSC frequency and amplitude (n = 8 from 6 animals, two-tailed unpaired Student’s t test). (d) The IF images showing co-label of GAD65&67 and SYN in dmPFC. Scale bar: 20 mm. (e) the quantification of GAD65&67^+^ and SYN^+^ synapse numbers. Statistical significance is exhibited as **P <0.05; **P<0.01; ***P<0.001; ****P<0.0001; ns, not significant*.
